# Continuous monitoring of the lower limit of reactivity in traumatic brain injury patients: understanding what is feasible

**DOI:** 10.1186/s13054-023-04773-3

**Published:** 2023-12-11

**Authors:** Erta Beqiri, Joseph Donnelly, Marcel Aries, Ari Ercole, Peter Smielewski

**Affiliations:** 1 Brain Physics Laboratory, Division of Neurosurgery, Department of Clinical Neurosciences, University of Cambridge, Cambridge, UK; 2 Department of Medicine, University of Auckland, Auckland, New Zealand; 3 School for Mental Health and Neuroscience (MHeNS), University Maastricht, Maastricht, The Netherlands; 4 Department of Intensive Care Medicine, Maastricht University Medical Center+, Maastricht, The Netherlands; 5 Division of Anaesthesia, Department of Medicine, University of Cambridge, Cambridge, UK

## Dear Editor,

A few months ago, we published our work ‘The Lower Limit of Reactivity as a potential individualised Cerebral Perfusion Pressure target in Traumatic Brain Injury: a CENTER-TBI High-Resolution Sub-Study Analysis’ [1]. We presented results of a validation study, where we investigated the relationship between the lower limit of reactivity (LLR) and the six-month outcome in a multicentre cohort of traumatic brain injury patients. We adopted a methodology that relies on the assessment of the vascular reactivity with the PRx index. The relationship between PRx and cerebral perfusion pressure (CPP) allows the identification of the CPP at a lower limit of cerebral autoregulation (CA) (or, in this case, cerebrovascular reactivity—LLR) for high levels of PRx at the lower ends of CPP. Using a multiwindow weighted approach, LLR can be estimated in a semi-continuous fashion (at a minute-by-minute resolution), providing means for individualising CPP treatment targets [2].

We are delighted to see that our work triggered reflection on the assessment of cerebral autoregulation in the critical care clinical practice and that Ayasse et al. raised the matter ‘Cerebral autoregulation: every step counts’ [3]. The authors question the methods used for the estimation of CA and the derivation of LLR. We must disagree with some of their comments and feel they may lead to confusion. We would like to take this opportunity to enrich the discussion on points we did not expand on in the main paper, as they were felt to be out of scope for that paper.

PRx is a well-established index of cerebrovascular reactivity in TBI patients, and it is supported by a large number of publications coming from different centres. Experimental studies have also validated PRx against cerebral blood flow (CBF)-based estimations of CA. Since vascular reactivity is the mechanism that enables CA, they have a link on a pathophysiological basis. Hence, there is merit in looking at the index PRx when it is potentially readily and continuously available, such as in TBI patients that require ICP monitoring, as no further monitoring devices would be required. The price to pay for such ‘simplicity’ is the fact that PRx is not a clean surrogate of CA and there are issues that require consideration.

Ayasse et al. correctly point out that the methodology of PRx-based CA assessment requires sufficient variability in arterial blood pressure (ABP). However, they then claim that such variability is limited in patients admitted in intensive care unit (ICU), due to ‘meticulous patient monitoring leading to accurate CPP targeting’. It is necessary to distinguish clearly between short-term and long-term variability in ABP. The variability required for PRx calculations is the latter: it is important to have enough variability (and thus power) from oscillations in the frequency range < 0.05 Hz within the 5-min data buffer used for each PRx calculations. Such variability is spontaneous and inherent to ABP, and it remains regardless of the management regime. Nonetheless, it is true that the short-term variability of ABP may sometimes not be sufficient to provide a good signal-to-noise ratio of PRx as exemplified by studies where PEEP-induced waves in ABP significantly decreased PRx variability [4, 5]. On the other hand, the long-term variability in ABP is under the control of the bedside clinical management. This, however, does not mean that ABP is kept stable, when viewed over hours. Furthermore, it is the combined, long-term variability of ABP and ICP, and thus CPP, that matters when attempting to trace and visualise the autoregulatory curve, required for ‘optimal’ CPP (CPPopt) or LLR calculations.

The nature of variability in ABP and ICP must also be considered carefully as pointed out by Ayasse et al. It is not really relevant whether the short-term variability in ABP is entirely internally driven (slow waves) or externally induced as long as the trigger does not impact ICP independently, violating implied causality. There are indeed episodes where the latter is true (suctioning events, position changes) which should be ideally eliminated from the data stream, along with other ABP and ICP artefacts [6], prior to calculation of PRx. However, when examined over 30–60 min or longer period of time, most of the artefactual effects would normally have limited influence on the average values. Also, the cases of ICP-driven ABP changes, which occur at extreme values of ICP, are not disconcerting as they correspond to scenarios of complete failure of CA, and thus PRx of close to +1, regardless. More worrying are effects such as described by Tas et al., where strong, synchronous waves in ABP and ICP were induced by the particular cyclic anti-decubitus mattress inflations, invalidating the PRx assumptions [7].

While PRx ranges between − 1 and + 1, setting a threshold for impaired autoregulation is a difficult matter. PRx reflects a relationship between the net blood volume change and their driving ABP variations. Contribution to this volume change likely comes mainly from the resistive arterial vessels, and these have a different reactive capacity depending on their size [8]. Furthermore, PRx estimates a global average vascular reactivity, while autoregulation may vary in different parts of the brain. Thus, discussing merits of one threshold over another, particularly taken from a small transitional range of values close to 0, is perhaps purely academic. In the literature, different values of PRx have been suggested as thresholds of lost pressure reactivity, based either on the ability of discriminating fatal and unfavourable outcomes in TBI patients [9], or on the ability of detecting the lower limit of cerebral blood flow regulation in experimental studies [10]. However, the vascular reactivity is still partially preserved at CPP levels below the lower limit of autoregulation [8, 11]. The threshold of 0.3 was considered by Donnelly et al. [12], on the premise that this would represent severely impaired global cerebral autoregulation. All in all, we did not aim to suggest a new threshold for the continuous assessment of LLR based on values of PRx in TBI patients, as this is not a simple on/off phenomenon. Instead, we aimed to validate the ability of the LLR approach in terms of outcome prediction. The fact that this method requires setting a particular threshold for the assessment of LLR, represents a substantial disadvantage when compared to the CPPopt methodology. CPPopt does not depend on any subjective value or threshold, as it is identified by the optimum of the U-shaped curve. However, this is also its weakness as the CPPopt point may well correspond to a highly positive PRx value (complete loss of reactivity), or alternatively come from a highly flat curve entirely composed of negative PRx values, suggesting that no particular adjustments to CPP is probably beneficial.

In the ideal scenario for CA monitoring, the ICU clinician would have access to continuous CBF measurements (in addition to ABP and ICP), would be able to induce sustained ABP variability in a safe manner and also have the ability to integrate measurements from several locations, providing much needed spatial resolution. This may perhaps be possible in time. Meanwhile, technologies like PRx and CPPopt/LLR are still able to provide valuable insights into the brain pathophysiology, but it is paramount that their advantages and limitations are well understood and that they are interpreted appropriately in order to be clinically useful.

## Data Availability

Not applicable.

## Abbreviations

TBI: Traumatic brain injury
CPP: Cerebral perfusion pressure
CBF: Cerebral blood flow
CA: Cerebral autoregulation
LLR: Lower limit of reactivity
ICP: Intracranial pressure
PRx: Pressure reactivity index
ABP: Arterial blood pressure
ICU: Intensive care unit
